# Biliary Atresia: 50 Years after the First Kasai

**DOI:** 10.5402/2012/132089

**Published:** 2012-12-06

**Authors:** Barbara E. Wildhaber

**Affiliations:** Division of Pediatric Surgery, Department of Pediatrics, University Hospital of Geneva, 1211 Geneva, Switzerland

## Abstract

Biliary atresia is a rare neonatal disease of unknown etiology, where obstruction of the biliary tree causes severe cholestasis, leading to biliary cirrhosis and death in the first years of life, if the condition is left untreated. Biliary atresia is the most frequent surgical cause of cholestatic jaundice in neonates and should be evoked whenever this clinical sign is associated with pale stools and hepatomegaly. The treatment of biliary atresia is surgical and currently recommended as a sequence of, eventually, two interventions. During the first months of life a hepatoportoenterostomy (a “Kasai,” modifications of which are discussed in this paper) should be performed, in order to restore the biliary flow to the intestine and lessen further damage to the liver. If this fails and/or the disease progresses towards biliary cirrhosis and life-threatening complications, then liver transplantation is indicated, for which biliary atresia represents the most frequent pediatric indication. Of importance, the earlier the Kasai is performed, the later a liver transplantation is usually needed. This warrants a great degree of awareness of biliary atresia, and the implementation of systematic screening for this life-threatening pathology.

## 1. Introduction to Biliary Atresia

Biliary atresia (BA) is a rare neonatal disease usually manifesting in the first months of life, when ascending obstruction of the biliary tree causes severe cholestasis and rapidly progressing biliary cirrhosis.

### 1.1. Classification and Anatomy

BA occurs most often (~90%) isolated without the presence of other malformations, but can also be part of a syndrome [1]. Syndromic BA can be associated with various congenital anomalies such as polysplenia or asplenia (100%), situs inversus (50%), preduodenal portal vein (60%), absence of retrohepatic inferior vena cava (40%), or cardiac anomalies (50%) [2].

BA is classified on anatomical bases, referring to the level and severity of the obstruction. The more commonly used Japanese and Anglo-Saxon classification describes 3 main types. In type I, atresia is limited to the common bile duct, and the gallbladder and hepatic ducts are patent (i.e., “distal” BA). In type II, atresia affects the hepatic duct, but the proximal *intrahepatic* ducts are patent (i.e., “proximal” BA). Type II is subgrouped in type IIa, where a patent gallbladder and patent common bile duct are present (sometimes with a cyst in the hilum, i.e., “cystic BA”), and in type IIb, where the gallbladder as well as the cystic duct and common bile duct are also obliterated. In type III, there is discontinuity of not only the right and left intrahepatic hepatic ducts, but also of the entire extrahepatic biliary tree (i.e., “complete” BA). The French classification is similar, but the designation of the above types IIa and IIb as types 2 and 3 results in a total of four types [3]. 

Most often, BA is complete (Japanese/Anglo-Saxon type III, 73%) or subcomplete (type IIb, 18%), with “cystic” BA and “distal” BA being infrequent (types IIa and I, 6% and 3%, resp.) [4]. 

### 1.2. Epidemiology

The reported incidence of BA in several European countries varies between 1/14,000 and 1/20,000 live births [4–8]. In the Pacific Ocean area, the incidence is described to be as high as 1/2,400 live births [4, 9–12]; reasons for this high incidence have not been described yet.

Some analyses of time and space distribution of BA cases suggested seasonal variation and clustering of cases [9, 13, 14], but these observations were not confirmed in larger studies [4, 7, 15, 16].

### 1.3. Etiology

The etiopathogenesis of BA remains unknown, although infectious, immune, genetic, and morphogenic origins have been invoked. 

In BA, damage most likely occurs during *early gestation*. In a series of 9 babies with prenatally detected biliary cystic malformation, only one patient had a polysplenia syndrome; all others had isolated BA [17]. Further, in a study where various gastrointestinal enzymes (specifically gamma-glutamyl transpeptidase) were measured in serial samples of amniotic fluid, there was definite evidence of bile obstruction *early* in the second trimester even in cases of nonsyndromic BA detected “incidentally” [18].

The role of an infectious process in the pathogenesis of BA has been extensively assessed. Hepatotropic viruses such as human papillomavirus [19], respiratory syncytial virus [20], herpes virus [21], cytomegalovirus (CMV) [22], reovirus type 3 [23], rotavirus [24], and Epstein-Barr virus (EBV) [25] have been implicated. In a Norwegian study of 10 consecutive BA patients, eight of 10 children and one parental couple presented laboratory results suggestive of recent or persistent viral infection, four by CMV and five by EBV, with detection of CMV-DNA in two liver biopsies and of EBV-DNA in one, with no sign of viral infection being detected in a control group of 10 patients matched by age and tested by PCR in serum and viral antibodies [26]. In a study where the amount and distribution of immunoglobulin deposits in liver biopsies from infants with BA were studied and correlated to the results to the CMV infection status, it was found that the intensity for IgM deposits was significantly higher in biopsies from patients with BA infected with CMV than in those without CMV infection, supporting the theory that a CMV infection may trigger immunologic mechanisms in the pathogenesis of BA [27]. It is plausible that a pre- or perinatal hepatobiliary viral infection might secondarily lead to an autoreactive T cell-mediated bile duct injury [28], whereby infection with a cholangiotropic virus results in initial bile duct epithelial damage, followed by persistent autoimmune-mediated inflammation and injury to bile duct epithelia despite clearance of the virus. Supporting this hypothesis, in murine models of BA, both cellular and humoral indicators of autoimmunity can be detected, and the progressive bile duct injury is due in part to a bile duct epithelia-specific T cell-mediated immune response [29].

Challenging the infectious hypothesis, a recent and extensive study failed to detect virus-specific sequences in liver samples from 74 BA patients analyzed by PCR, and the overall virus incidence of 42% failed to verify the hypothesis of a viral etiology of BA. Furthermore, the PCR-virus incidence in patient with and without coincidental malformations was similar, which again argues against a specific role of viruses in the nonsyndromic form of BA [30]. 

Even though BA is not believed to be an inherited disease, genetic factors are thought to play a role in its pathogenesis. There are reports of familial cases of BA [31–33], yet, discordant sets of monozygotic twins have also been observed [34–36]. The genetic theory is supported by the observation of increased incidence of BA in Polynesia or Asia [4, 9–12], with different incidences amongst groups with distinct genetic background. Genetic factors are most probably involved in the BA polysplenia syndrome, as a recent French study found an association between heterozygous CFC1 mutation, left-right laterality defects, and BA with splenic malformation [37]. Furthermore, in the mouse model, genetic inactivation of hepatocyte nuclear factor 1 beta resulted in morphological anomalies in intrahepatic bile ducts and in the gallbladder [38], suggesting that mutations in genes that regulate hepatobiliary development may play a role in BA. 

### 1.4. Diagnosis

#### 1.4.1. Prenatal Diagnosis

BA may be suspected prenatally, when a cystic structure is observed in the porta hepatis [39]. In that case further investigations must be performed rapidly after birth, in order to distinguish a choledochal cyst, which does not require immediate intervention, from the cystic form of BA, which calls for urgent surgical treatment (see below). 

#### 1.4.2. Clinical Features

**Figure 1:**
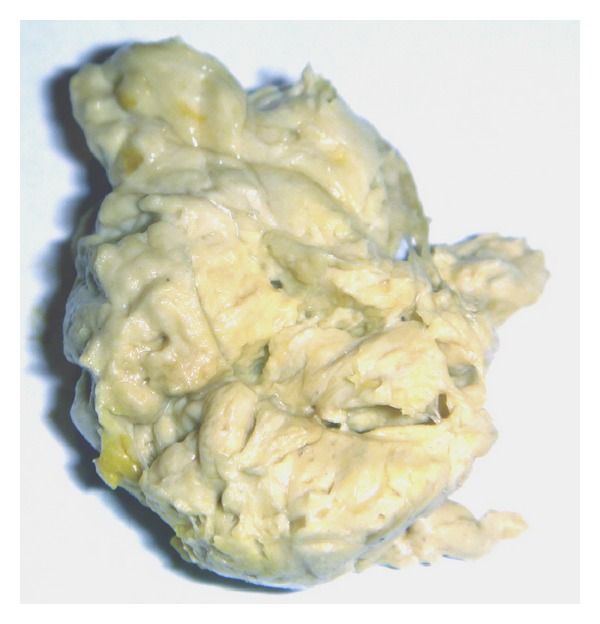
Acholic, discolored stool of a baby with biliary atresia.

Postnatally, the classic clinical triad of BA is constituted by (i) jaundice (conjugated, and persisting beyond two weeks of life), (ii) acholic (white) stools (Figure 1) and dark urine, and (iii) hepatomegaly. All cases of neonatal jaundice lasting longer than 14 days *must* be explored in order to rule out BA or other causes of neonatal cholestasis [40–42]. At an *early* stage the baby exhibits no failure to thrive, is well nourished, and is in good condition. However, the *later* the diagnosis, the more BA-associated clinical signs appear, such as splenomegaly and ascites, which both suggest portal hypertension, and intracranial or gastrointestinal hemorrhage, due to either portal hypertension or impaired vitamin K absorption. 

#### 1.4.3. Laboratory Studies

In cases of BA, biochemical liver function tests are typically those of cholestasis, with elevated levels of total and conjugated bilirubin (>20 umol/L total bilirubin, of which >20% is conjugated), increased gamma-glutamyl transpeptidase and alkaline phosphatase, and sometimes slightly higher than normal transaminases [43]. 

#### 1.4.4. Imaging Studies

Abdominal ultrasonography is the first choice and gold standard noninvasive imaging investigation when BA is suspected. It needs to be performed after 8 to 12 hours fasting, with BA being suspected if the gallbladder is shrunken despite the absence of feeding, if the liver hilum is hyperechogenic (“triangular cord sign”), or if there is a cyst at the liver hilum without bile duct dilatation [44]. Polysplenia, a preduodenal portal vein, or absence of the retro-hepatic vena cava may be found in syndromic BA babies. Note again that approximately 20% of BA cases have a patent gallbladder. 

Hepatobiliary scintigraphy using technetium-labeled iminodiacetic acid derivatives aims at offering a dynamic, objective assessment of both parenchymal liver function and biliary excretion. In BA this exam will reveal a lack of excretion of the radioisotope into the intestine; however, this can also be observed in any severe neonatal nonobstructive cholestasis [45]. 

Magnetic resonance cholangiopancreatography (MRCP) is increasingly performed in cases of neonatal cholestasis, as it allows for a visualization of the major biliary structures, hence for the exclusion of BA [45]. In a Korean study the diagnosis of BA was made by MRCP based on the nonvisualization of extra-hepatic bile ducts and excluded on the basis of the complete visualization of extra-hepatic bile ducts; accuracy was 98%, with sensitivity and specificity of 100% and 96%, respectively [46]. Nevertheless cholangiography still remains the gold standard to diagnose BA, as described below. 

#### 1.4.5. Invasive Studies

When BA cannot be formally excluded, especially when a gallbladder is present, a cholangiography is needed to assess the morphology of the biliary tree, and ascertain that there is patency between the liver and the intestine. The cholangiogram can be obtained percutaneously [47], by laparoscopy [48], via an open laparotomy, or through an endoscopic retrograde cholangio-pancreatography (ERCP) [49–51]. 

The other most definitive test for establishing the diagnosis of BA is a liver biopsy, usually performed at the same time as the cholangiography. The main features suggesting BA are biliary tracts containing inflammatory and fibrous cells surrounding miniscule ducts, likely remnants of the original ductal system, with a fibrotic liver parenchyma exhibiting signs of cholestasis, and proliferation of biliary neoductal structures [52].

### 1.5. Differential Diagnosis

Alternative causes of neonatal cholestasis need to be excluded in order to distinguish BA and other anatomical origins from their functional counterparts, that is, diseases that require surgical intervention from ones that can be managed medically [53]. The most common differential diagnoses of BA are Alagille syndrome, Progressive Familial Intrahepatic Cholestasis (PFIC), alpha-1-antitrypsin deficiency, and cystic fibrosis. If these other pathologies are ruled out, particularly when the gallbladder seems normal on ultrasound, a cholangiography and liver biopsy absolutely need to be performed [54]. 

## 2. Surgical Management of Biliary Atresia

The current management of BA patients is sequential and involves two steps: (1) Kasai hepato-porto-enterostomy (HPE) in the neonatal period, in order to restore bile flow towards the intestine and preserve liver function as long as possible; (2) liver transplantation (LT), if no clearance of jaundice can be achieved through the Kasai operation or when complications of biliary cirrhosis appear later on. 

The Kasai HPE remains the preferred initial treatment, even though it is estimated that about 80% of patients with BA will ultimately need a LT. In a French study where 271 patients were analyzed, only 23% were alive with their *native* liver twenty years after a Kasai [55]. Yet, the later in life the transplantation must be performed, the lower its morbidity and mortality [56]. 

### 2.1. Kasai Hepatoportoenterostomy

The Kasai aims at restoring bile flow between the liver and the intestine, using a jejunal Roux-en-Y limb, which is anastomosed to the porta hepatis after resection of the biliary remnant. This hepato-porto-enterostomy is named after the Japanese surgeon Morio Kasai, who first performed this procedure in 1959 [57]. 

**Figure 2:**
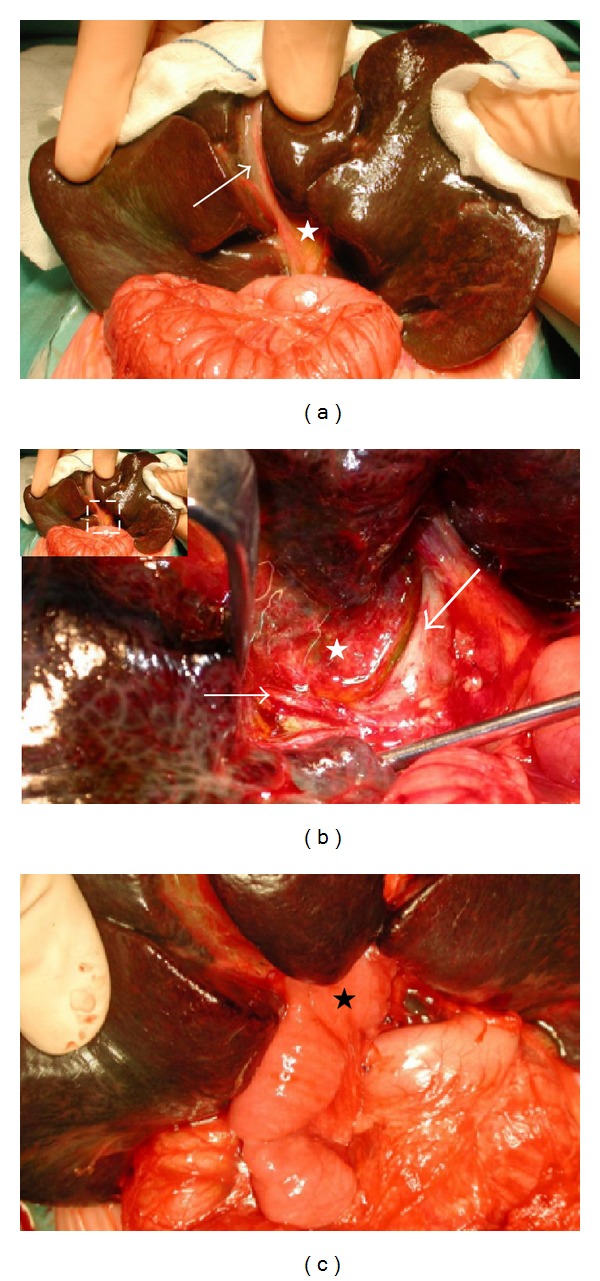
(a) Complete biliary atresia with a shrunken, fibroticgall bladder (arrow) and biliary remnant (white star) in a 2-month-old baby. Note the already cirrhotic aspect of the liver. (b) Hilar region after removal of the biliary remnant (white star), surrounded by the hepatic artery's right branch (fine arrow) and the portal vein's left branch (large arrow). (c) Final aspect of the Kasai hepato-porto-enterostomy with the Roux-en-Y limb (black star) anastomosed to the hilar region.

At the beginning of the operation the diagnosis of BA is confirmed by inspection of the liver and biliary tract, usually through a small transverse upper abdominal incision at the level of the 11th rib (see Section 2.3). In most cases the diagnosis is obvious when a cholestatic or even fibrotic liver and a shrunken, fibrotic gallbladder are found. If the gallbladder is patent, or if there is a cyst at the liver hilum, a cholangiography must be performed. The color of the cyst's or gallbladder's content must be noted (yellow-green versus transparent), as it gives information about the type of BA and therefore the kind of surgical approach to be used (see Section 2.4). When BA is confirmed, the transverse abdominal incision is enlarged to approximately 12–15 cm, 1/3 to the left of the umbilicus, and 2/3 to its right. For easy access to and best visualization of the liver hilum the skin incision is placed behind the liver by lifting the organ forward, without need for transecting the falciform or triangular ligaments (Figure 2(a)). Note that if the incision is too large, the skin will not hold back the liver and prevent it from sliding back into the abdomen. To ensure a good venous backflow, no padding should be placed behind the baby's lower thorax at the time of its installation for the operation, as usually done to expose the hilar region. Upon inspection of the abdominal cavity, attention must be paid to other possible anomalies, in particular those associated with the polysplenia syndrome. If a Meckel's diverticulum is present, it should be resected, since it increases the risk of major bleeding later in life, if portal hypertension develops. In the case of a standard Kasai HPE the gallbladder remnant is mobilized from its bed and the biliary remnant is meticulously dissected above the portal vein bifurcation towards the periphery, where the portal vein as well as the hepatic artery divide into their branches towards the right and left liver lobe. To obtain a better visualization of the left extension of the biliary remnant, the bridging hepatic parenchyma, surrounding the round ligament between the segment 3 and 4, must be divided. The fibrotic common bile duct is transected distal to the remnant of the cystic duct. Once the entire remnant is properly prepared, it is removed from the liver bed with scissors, just at the level of the liver parenchyma, which will appear as a slightly whitish surface (Figure 2(b)). If the remnant is cut too deeply into the liver parenchyma, scarring may occur and initially opened biliary ductules may close up again secondarily. On the contrary, if the remnant is cut too superficially, the lumen of the biliary ductules will not be exposed and bile flow will not be restored. The success of the operation remains critically dependent on the meticulous dissection of the liver hilum and the careful resection of the biliary remnant. This dissection is followed by cautious anastomosis of the antimesenterically opened bordure of the 45 cm long intestinal Roux-en-Y limb, which is passed through the mesocolon, laterally and anteriorly to the duodenum (Figure 2(c)). The anastomosis can be performed with continuous or interrupted sutures, usually with a 6/0 resorbable, monofilament material, paying attention not to occlude any lateral bile ductules in the periphery of the hilum. No drainage tube is necessary. 

Clearance of jaundice is achieved in about 50 to 60% of cases (see Section 5) [58–61], when using this conventional technique.

### 2.2. Modifications of the Traditional Kasai Procedure

#### 2.2.1. Wide Dissection

With the objective of achieving better results, that is, longer survivals with the native liver, several modifications of the traditional Kasai HPE have been described (Table 1). Most reports emphasize the importance of wide dissection, in order to free small peripheral biliary ductules [62–65], or even suggest dissection into the parenchyma of liver segment 4 and more to open up dystrophic ductules in the liver [66]. For instance, in 1997, Hashimoto et al. propose to extend the dissection to the left up to the umbilical point (segmental branches 1, 2, 3, and 4 on the left side of the hepatic hilum), and to the right towards the dorsal aspect of the anterior portal branch *over* the bifurcation of the anterior and posterior segmental portal vein branches. Then, the hepatic parenchyma at the hepatic hilum, including segment 4, is partially trimmed with the Cavitron ultrasonic suction aspirator (CUSA) to isolate every segmental biliary remnant, albeit without applying the CUSA directly to the remnant. Before removal of the remnant, five stitches for traction are then placed on each segmental remnant, and the fibrous degenerated extrahepatic bile duct at the porta hepatis is removed from the liver parenchyma using knife and scissors [66]. In a series of 39 patients treated by this approach, a remarkable 77% became completely and continuously free of jaundice, without need for LT or reoperation [66]. The placement of some interrupted holding stitches into the liver surface on the posterior side of the remnant fibrous mass (just above the portal bifurcation) before transection, in order to expose the remnant better, was also demonstrated as beneficial in another Japanese series of 14 cases, 13 of whom became anicteric [67]. In 2010 Suzuki et al. described a similar but less extensive trimming of the liver, again using the CUSA, with, again, exteriorization of the liver from the abdomen dissection at the porta hepatis with systematic exploration of the extrahepatic biliary remnant using the portal vein as a landmark, and CUSA-mediated trimming of the hepatic parenchyma restricted to segment 4, just enough to expose every segmental biliary remnant [68]. Of the 53 patients of this series a spectacular 81.1% returned to normal total bilirubin levels, albeit sometimes after as long as 7 months after surgery, whereas the outcome of a conventional Kasai is usually determined at 3 months after HPE [68]. Of note, in this study, high doses of steroids were administered in parallel to the surgery, which may have influenced the outcome. In 1997, Ando et al. reported on their attempt to further expose the biliary remnant by dividing the ligamentum venosum [69]; after a conventional approach to dissect the biliary remnant along the hilar vessels, several fine portal branches to the left and right caudate lobe are ligated and divided. The ligamentum venosum (Arantius' canal), branching from the left portal vein at the bifurcation of the lateral segment and the umbilical portion, is then also ligated and divided, thus mobilizing the portal vein and exposing the porta hepatis more fully. The fibrous cord of the porta hepatis can thus be easily dissected off posteriorly and laterally, where extensive numbers of bile ducts are present. Finally, the Roux-en-Y limb can be anastomosed both to the caudate lobe posteriorly and to the quadrate lobe anteriorly [69]. In a series of six patients treated by this procedure, jaundice resolved completely in all within 40 days [69]. It remains that Hashimoto et al. strongly suggested never to mobilize completely the portal vein, as it increases the risk of injuring its small branches [66]. This and other debates illustrate the fact that the work on the optimization of the traditional Kasai procedure is still in progress, and that no gold standard has been defined over the years—other than the general approach of removing the biliary remnant and anastomosing an intestinal limb to the liver hilum. 

#### 2.2.2. Prevention of Ascending Cholangitis

Postoperative cholangitis, due to contamination of biliary ductules by the intestinal microbial flora, is most prevalent early after Kasai HPE [70], and appears to influence strongly the outcome [71–74]. Accordingly, several operative innovations have been presented in the past, mainly by Japanese surgeons, to prevent stasis in the Roux-en-Y limb and reflux to the porta hepatis (Table 1).

**Antireflux Valve**

An intussusception antireflux valve in the Roux-en-Y limb of the HPE has been advocated since the late seventies to prevent postoperative cholangitis [75–77]. An antireflux valve can be created by dividing the mesenteric blood vessels over a 4 cm length of the Roux-en-Y loop, denuding further the distal 1.5 cm from the seromuscular layer, and invaginating the proximal portion into the denuded jejunum [75]. In a series of 23 children treated by this procedure, Nakajo et al. reported in 1990 the absence of ascending cholangitis in any of 17 new cases with BA during an average followup of 32 months [75]. This procedure was subsequently used in BA patients suffering from recurrent cholangitis after a Kasai, with significant decreases in the incidence of infections [78]. In contrast, Ogasawara et al. performed in 2003 a prospective study where the incidence of postoperative cholangitis was compared in between groups of patients with and without valve. The total number of patients who had cholangitis as well as the total number of episodes of cholangitis and the mean duration of these episodes were not different between the two groups, casting doubt on the benefit of an anti-reflux valve to prevent such complication in the early post-HPE period [79].

**Enteric Conduit**

Kasai soon modified his technique (to the so-called Kasai II operation) by introducing a double-Y, where the Roux-en-Y limb is transsected below the hepatic anastomosis and this proximal segment is then exteriorized through the abdominal wall; the distal segment is anastomosed to this proximal segment in an end-to-side fashion, so that the diversion is not complete [80]. In 1968 a complete external fistula was suggested by Sawaguchi et al., where the entire Roux-en-Y is exteriorized as a jejunostomy [81], which appeared to decrease the incidence of cholangitis [82, 83]. In 1977 Suruga et al. suggested to perform a double-barrel ostomy of the Roux-en-Y limb [84], again with results consistent with a lower incidence of postoperative cholangitis. Another technique to prevent ascending cholangitis was described by Endo et al. in 1983, whereby an ileocolic conduit of 30 cm of distal ileum is anastomosed to the porta hepatis and a 10 cm segment of ascending colon is vented through the abdominal wall, thus including the ileocecal valve [64]. This technique was extended in 1995, with secondary anastomosis of the previously excluded part of the colon to the second portion of the duodenum, one year after the HPE [85]. The authors of this study concluded that the ileocolic conduit led to a decreased frequency of ascending cholangitis and—surprisingly—improved the patient survival and quality of life, although some metabolic and physiological drawbacks were noted [85]. Of interest, the draining bile in the case of the colonic stoma was highly concentrated due to the water-absorbing capacity of the interposed colonic segment; therefore, fluid and electrolyte disturbances, which develop frequently in patients having jejunal conduits, have not been encountered in this technique [64]. Of note, all these different anticholangitis techniques imply a second operation when the ostomy is suppressed, one to two years after HPE, that is, when the risk of cholangitis starts decreasing. In 2001 Liu and Li compared the two anti-cholangitis techniques. Twenty-four patients with BA underwent HPE with percutaneous jejunal enterostomy and 24 patients underwent Roux-Y with anti-reflux valve; the two surgical procedures had similar effects in preventing reflux cholangitis, while the valve obviously had the benefit of avoiding a cutaneous enterostomy [86].

It is noteworthy that none of these anti-cholangitis modifications ever gained broad acceptance in the community of pediatric surgeons, as their benefit remains questionable and they may be the source of complications by causing important hydroelectrolyte losses through the stomas, and major blood losses in case of portal hypertension and beginning liver failure [56]. 

### 2.3. Laparotomy versus Laparoscopy

The HPE is usually performed via a transverse laparotomy but can also be achieved using laparoscopy [87, 88], in particular with robotics [89]. Yet, recently discussions have emerged about the evidence-based indication of the laparoscopic Kasai operation. In 2011 Ure et al. reported a prospective study that demonstrated that this approach is indeed technically feasible, but the study was stopped after inclusion of 12 infants subjected to a laparoscopic Kasai, due to a significant lower survival with the native liver compared to children treated by its laparotomic counterpart [90]. Another study failed to detect any benefit of laparoscopic compared with conventional HPE, in particular no lower incidence of adhesion formation hence easier subsequent LT, one of the intended goals of the laparoscopic approach [91]. Accordingly, some clinics have completely abandoned the laparoscopic approach for a Kasai. 

### 2.4. Technical Variants of the Kasai Procedure

It must be remembered that whatever the type of BA, the classical Kasai HPE is always the right choice of operation. Yet, several technical variants of the traditional Kasai HPE are possible, depending on the anatomical pattern of the biliary remnant. In case of a patent gallbladder with patent proximal bile ducts (i.e., type I or “distal” BA), a cholecystoenterostomy or hepaticoenterostomy can be performed; in case of the cystic form of BA, where the cyst communicates with the intrahepatic bile ducts, a cystoenterostomy may be an option, and in case of patent gallbladder, cystic ducts and common bile duct (i.e., distal BA), a hepatoportocholecystostomy may be carried out, whereby the gallbladder with its artery is mobilized and anastomosed to the nude liver surface of the porta hepatis from where the biliary remnant was removed. Using this technical variant, with no direct contact between the porta hepatis and the intestine, the risk of postoperative cholangitis is reduced [92]. Yet, bile leakage with postoperative biliary ascites due to kinking and obstruction of the cystic and common bile duct is a complication specifically associated with this technique [93, 94]. 

### 2.5. Postoperative Care

If the Kasai operation succeeds, bile flow is restored, the stools become colored, and jaundice fades. Thus, the natural evolution of BA towards biliary cirrhosis is delayed or even, rarely, altogether prevented, so that the Kasai operation may help to postpone LT until late childhood, adolescence, or even adulthood [8].

What is the best postoperative management after HPE remains unestablished. Different drugs may be administered, but (gold) standardized protocols have not been defined. Prophylactic antibiotics to prevent cholangitis [95, 96], barbiturate, cholestyramine, and ursodeoxycholic acid to enhance bile flow [96, 97], and steroids to reduce inflammation might be beneficial. Glucocorticoids stimulate the transcription of genes coding for anti-inflammatory proteins [98] and, additionally, steroids appear to have positive pharmacological actions on the bile flow [99, 100]. Yet, the use of this category of drugs remains controversial. Postoperative steroids have been used empirically for many years, with a number of retrospective, uncontrolled reports suggesting a benefit [101–104]. A recent randomized, double-blind, placebo-controlled trial of prednisolone after Kasai HPE (initial dose 2 mg/kg/day) indicated a beneficial effect on the rate of reduction of bilirubin in the early postoperative period (specifically in infants less than 70 days old at surgery), but no reduction in the ultimate need for LT [105]. Currently a large-scale double-blind, prospective study is ongoing in the USA, with a high-dose regimen (initial dose of prednisolone 5 mg/kg/day), the results of which are expected within a few years. 

## 3. Complications of Biliary Atresia 

### 3.1. Surgical Complications

Surgical complications are rare after a traditional HPE [106]. They comprise the standard complications of abdominal surgery, including adhesive ileus, wound dehiscence, anastomotic intestinal leak, or intussusception at the foot point of the Roux-en-Y limb, as well as internal hernia [107]. Bleeding from the porta hepatis or bile leakage from the hilar anastomosis is specific to the HPE but, again, is rare [108]. 

### 3.2. Postsurgical/Postmedical Complications

The majority of complications in BA patients is medical. Without exception they are severe and often represent indications for LT. 

#### 3.2.1. Cholangitis

The baby is particularly prone to ascending cholangitis during the first weeks after the operation, and ascending cholangitis occurs in 30–60% of cases [70]. This infection may be severe and sometimes fulminant. Clinically there are signs of sepsis, recurrence of jaundice, acholic stools, and perhaps abdominal pain; blood cultures may be positive. Treatment consists of IV antibiotics during two to three weeks. Recurrent cholangitis may require continuous antibiotic prophylaxis. The number of cholangitic episodes has been shown to negatively influence the success of the Kasai operation [71–74]. It has been reported that the occurrence of cholangitis significantly reduces survival rate in patients with either good or inadequate bile flow [73]. The more frequent the episodes of cholangitis are, the greater the probability of cirrhosis and the poorer the outcome of the HPE [71, 74]. 

#### 3.2.2. Portal Hypertension

Almost half of BA patients present with bridging fibrosis at the time of the Kasai HPE [74]. This finding, associated with a higher portal pressure, is not only highly related with a lower chance of success of the operation, but also a higher risk of developing portal hypertension [109]. 

As the disease is progressive, all children with BA eventually will develop portal fibrosis, cirrhosis, and portal hypertension to some extent, even if bile drainage has been established [96]. The most common sites of varices are the esophagus, the stomach, the jejunal site of the Roux loop anastomosis, and the anorectum. In case of portal hypertension, associated with progressive liver failure and/or persistent jaundice, LT is indicated. Yet, local treatment of oesophageal varices may be needed prior to LT, using endoscopic variceal sclerotherapy or band ligation [110]. If liver function is preserved and jaundice is absent, endoscopic therapy may be sufficient [111]. Surgical portosystemic shunts may be considered if liver function is still normal and there are no life-threatening varices [112]. Transjugular intrahepatic portosystemic shunts (TIPS) are rather rarely used in BA patients; the procedure is technically difficult, mainly due to periportal fibrosis and, typical for BA, small portal veins. But a TIPS can temporarily help in the management of portal hypertension in children, especially in those needing momentary relief before LT [113]. 

#### 3.2.3. Hepatopulmonary Syndrome and Pulmonary Hypertension

As in any patient with severe chronic liver disease, where portosystemic shunts occur due to portal hypertension, a hepatopulmonary syndrome may develop, associated with intrapulmonary arteriovenous shunting, characterized by hypoxemia, cyanosis, and dyspnea, in the absence of primary cardiac or pulmonary disease. While the physiopathology of this condition is not formally established, it is likely influenced by gut-derived vasodilative agents such as nitric oxide, glucagon, and platelet activating factor, which enter the systemic circulation through the portosystemic shunts, avoiding clearance by the liver [114, 115]. The hepatopulmonary syndrome, a complication typically encountered in the long-term followup of children with BA, may represent an indication for early LT even when liver function is preserved [116].

The same general physiopathological mechanisms may lead to the development of a vasoconstrictive/obliterative process, affecting the pulmonary arterial bed, causing increased pulmonary vascular resistance, described as portopulmonary hypertension. It can lead to sudden death [117]. Moderate to severe portopulmonary hypertension has been associated with significantly increased perioperative morbidity and mortality during LT, primarily due to right ventricular failure immediately after reperfusion [118].

LT usually suppresses pulmonary shunts [119, 120], but pulmonary hypertension is reversible only at its early stage [117]. 

#### 3.2.4. Intrahepatic Biliary Cavities

 Intrahepatic biliary cysts, solitary or multiple, may appear even after a successful Kasai operation in about 20% of cases [121]. Some of these cysts can contribute to recurrent cholangitis and affect morbidity and mortality. Treatment, particularly in cases complicated by cholangitis, consists of percutaneous transhepatic cholangiodrainage [121], as well as, less frequently used, local alcohol injections [122], or, in cases where permanent drainage is necessary, internal intestinal drainage with a cystenterostomy [123]. In severe cases it may be an indication for LT.

#### 3.2.5. Malignancies

Liver cirrhosis can be further complicated by malignancies such as hepatocellular carcinoma [124, 125], hepatoblastoma [126], and cholangiocarcinoma [127, 128], all reported in BA patients, whether in childhood or in adulthood. Screening of alpha-fetoprotein and ultrasonographic examination should be performed regularly in BA patients with secondary biliary cirrhosis for early detection of tumor formation. 

## 4. Liver Transplantation for Biliary Atresia

If the Kasai operation is not successful, that is, no restoration of bile flow is achieved, and/or medical complications of biliary cirrhosis appear, even if jaundice has subsided, LT is indicated. Most transplantations for BA patients are performed in the first or second year of live. BA represents about half of the indications for LT in childhood [129]. LT should not be deferred too long once it becomes apparent that it will be required, since timing of LT not only affects survival, but may also influence neurodevelopmental outcome [129]. Nutritional support before transplantation and, reciprocally, performance of transplantation before malnutrition develops may reduce developmental delays [130].

There are two sources for a liver graft. (1) *Cadaveric donor*: the graft usually derives from an adult donor, and the left lobe (segments 2 and 3) or the left liver (segments 2, 3, and 4) is used after *in situ* or *ex situ* splitting of the whole liver. Pediatric donors are much less frequent, but in those cases the whole liver can be transplanted. (2) *Living-related donor*: usually the donor is a close relative, in most cases one of the parents. A left lobectomy is generally performed to transplant segments 2 and 3. Morbidity of the lobectomy performed in the donor is not negligible, reaching 10% [131, 132]. 

The goal of the LT is to offer a normal life to these children, allowing for normal physical, intellectual, psychological, sexual, and social development [133]. 

## 5. Outcome of Biliary Atresia Patients

Before the Kasai operation was developed, most children with BA died before the age of two. The worldwide diffusion of the Kasai HPE in the 1970s considerably changed the prognosis of this disease, yet many children still died from the complications of biliary cirrhosis. Only after the introduction of LT in the 1980s did the outcome of BA patients dramatically improve, so that survival now reaches 90% in industrialized countries [134]. 

### 5.1. Survival with Native Liver

In Western countries short-term clearance of jaundice can be achieved with the Kasai HPE in approximately 50 to 60% of children [58–61]. This is closely related to the widely used outcome measure for BA patients, that is, the survival with native liver (SNL). Four-year SNL was described to be 48% in France [135], 51% in the 3 supraregional British centers (Birmingham, Leeds, London) [58], 49% 5-year SNL in Madrid (Spain) [136], 37.4% in a Swiss national series [137]. When SNL is compared with the overall patient survival (i.e., survival with Kasai and/or LT) it is seen that among the patients alive at the age of four years, 60% have been transplanted in Switzerland, 51% in France [135], and 43% in the United Kingdom [58]. As a general rule, half of BA patients need a LT in the first two years of life [137], one-third of patients can survive with their native liver up to the age of ten years [55, 138, 139], and one-fourth up to twenty years [55, 140]. Yet, even if some patients treated for biliary atresia will survive into adulthood with their native liver, they will commonly present with secondary biliary disease including cholangitis and portal hypertension [141]. 

### 5.2. Overall Survival

Overall survival of BA patients (i.e., patients after Kasai only, or Kasai and subsequent LT, or primary LT) has dramatically improved since the implementation of pediatric LT. The first series of pediatric LT was reported in the late 1970s [142], and the procedure has made tremendous progress since then. Not only surgical innovations but primarily medical advances nowadays allow a most positive prognosis for pediatric patients. This evolution is seen in the French national studies, where overall 5-year survival increased from 70% in the 1986–1996 study [3] to 88% in the 1997–2002 study [135]. In a UK series between 1999 and 2002, 89% of 148 patients survived four years and longer [58]. In the BARC (Biliary Atresia Research Consortium) series (104 patients from nine US centers, 1997–2000) 2-year overall survival of BA patients after Kasai operation and/or LT was 91.3% [143]. 

## 6. Prognostic Factors

Several prognostic factors of the Kasai operation have been related to the short-term results of this procedure. Among them are many that are *nonmodifiable*, such as (1) anatomy of the biliary remnant, that is, type of BA, (2) histology of the liver at the time of HPE, (3) portal pressure at the time of HPE, (4) association of BA with polysplenia syndrome. Other prognostic factors of BA are related to the organization of care for these patients and therefore are *improvable*: (1) experience of the center in the management of BA patients, (2) age at HPE, (3) accessibility to LT. 

### 6.1. Nonmodifiable Risk Factors

More favorable situations encompass a patent gallbladder and/or cystic dilatation of the extra-hepatic bile duct (type 2), or BA restricted to the common bile duct (type 1) [3, 60, 144]. Bridging fibrosis at the time of the Kasai HPE is associated with worse outcome [74, 145, 146]. BA patients with elevated portal pressure at the time of Kasai operation (>15 cm H_2_O) have lower chances of success of this procedure and a higher risk of developing portal hypertension, even if bilirubin levels are normalized after surgery [109]. Polysplenia syndrome is also associated with a worse prognosis [3, 92, 135, 143, 144]. Postoperative clearance of jaundice is a strong indicator of success for the Kasai HPE and thus predictive of the timing at which LT will be required [147–149]. Cholangitis is known to complicate the outcome of the Kasai HPE [71, 73, 74, 147, 150], and every episode needs early and vigorous therapy. 

### 6.2. Modifiable Risk Factors

#### 6.2.1. Case Load

As with other challenging operations, the case load of the center performing HPE seems to influence greatly the outcome of the intervention, that is, SNL. In the United Kingdom, two studies [8, 59] revealed a wide variation in SNL according to the experience of the centers in the management of BA patients. These findings led to the centralization of all British BA patients in 3 pediatric liver units (for a British population of about 60 million people), which are able to manage the child from diagnosis to LT. This policy proved to be efficient, with high level results being nowadays obtained for all children nationwide [58]. Davenport et al. showed in their UK experience that in the early nineties, before the centralization, clearance of jaundice was 44% and increased to 57% in 2002 after centralization [58]; a result that was confirmed by a larger, more recent study [61]. In France (same population of about 60 million people), a similar discrepancy of results was observed according to the centers' case load [3]. This led to the promotion of an increased collaboration between centers to standardize procedures and optimize results at the highest possible level [135]. In Finland, exceptional improvement was observed after centralization from five to one center; clearance of jaundice rate increased from 27% to 75%, 2-year jaundice-free native liver survival from 25% to 75%, transplant-free survival from 27% to 75%, and overall survival from 64% to 92% [151]. Following the evidence of these findings, in Switzerland (population of 8 million people), all BA patients have recently also been centralized in one national center. 

#### 6.2.2. Age at Kasai

The age of the patient when the HPE is performed has been repeatedly demonstrated to influence SNL in large series; short-term results of the Kasai are better when it is done early [3, 11, 42, 135, 137, 149, 150, 152–154], that is, at the latest by the end of the third month of life, with clear evidence that the earlier the operation, the better the outcome (see below). Syndromic BA, that is, “developmental” BA, exhibits an even worse outcome if operated on late [155]. Although some reports failed to establish a parallel between early intervention and success of the Kasai HPE [8, 74, 82, 156], their conclusions were likely flawed owing to insufficient numbers of patients [8]. Nevertheless, HPE can still be beneficial after 3 months of age; in the French 1986–1996 national series, still 25% of the patients operated after 3 months were alive with their native liver at 5 years [157].

Most of the reports studying early age at HPE look at short-term SNL, that is, 4- or 5-year SNL. Yet, a 2007 national Canadian study reported that the benefit of an early Kasai operation, in the first month of life, is maintained until late childhood and adolescence [158]. In 2009 Serinet et al. confirmed in a large French national study that the earlier the BA baby is operated on, the later LT will be required, with an effect maintained late into adolescence [159]. These authors evaluated the potential benefits of neonatal BA screening; children operated before 6 weeks of life were compared to those older; a 12.1% improvement in 15-year SNL (34.9% versus 22.8%) was measured following early intervention, clearly confirming that the earlier the diagnosis of BA, the later LT is required [159]. 

## 7. Newborn Screening

In order to improve outcome of BA patients, that is, to reduce the need for early LTs in these children, and last but not least, to save nonnegligible resources of the national public health system, measures must be taken towards an early diagnosis of BA. If a BA child can be maintained in good health for longer with its own liver, that is, if LT can be delayed after infancy and early childhood, it not only lessens the risks of the LT procedure itself, but also the frequency of medical complications often met in childhood, such as Epstein-Barr virus primary infection in the immunosuppressed child, the ground for a life-threatening Posttransplant Lymphoproliferative Disorder [160]. If pediatric LTs were prevented, this would also lead to significant financial savings; theoretical calculation showed that in the USA the yearly financial economies would amount to approximately 18 million dollars [159]. The cost of a widespread BA screening program would most probably never reach this sum, so that it would be a sound investment for society. 

Many screening programs for BA have been proposed, such as early measurement of serum bile acid [161], serum direct bilirubin [162], serum Apo C-II and III proteins [163], urinary sulfated bile acid [164], and fecal bilirubin and fat [165]; however, none has been put into practice extensively, due to both cost and technical complexity. A much simpler method, based on the detection of neonatal cholestasis through examination of the baby's stool color, represents an extremely attractive alternative; pale grey-pigmented stool is readily identified in 95.2% of children with BA in early infancy [166]. Such screening, which can rely on the use of a very simple stool color card, is easy and inexpensive. Both the parents and the pediatrician can easily detect pathologic stool pigmentation by confronting the baby's feces with color indicators on the card; an examination that optimally should be performed during the first month of life in order to have enough time for confirming the diagnosis and performing an early HPE. The first concept of routine screening of newborns for BA using a stool color card was initiated in Japan in the early 1990s [167, 168], introduced nationwide in Taiwan in the early 2000s [12, 169, 170], and is now also available in Switzerland [171]. In Taiwan, five years after introduction of the systematic stool color card screening, the rate of the Kasai operation before 60 days of life increased from 49.4% to 65.7%, the jaundice-free rate at 3 months after HPE from 34.8 to 60.8%, and the 5-year SNL from 27.3% to 64.3%; all are significant positive changes [172]. The stool color card was thus proven to be a simple, noninvasive, efficient, low-cost, and applicable mass screening method for early diagnosis and management of BA, hence an ideal mean to help identify a devastating disease, that, if not treated early in life, inexorably leads to the need to overly precocious and risky LT in infancy, secondarily depriving the community of most precious organs for transplantation. The benefit of this BA screening program is thus not only paramount for the child and his family, but also for society at large. 

## Figures and Tables

**Table 1:** Modifications of the traditional Kasai hepato-porto-enterostomy.

	Wide dissection of the biliary remnant
	*Hashimoto modification*: Extension of the lateral dissection into segmental bifurcations of the portal vein, followed by trimming the liver with the CUSA [66]
	*Suzuki modification*: Trimming of the segment 4 of the liver with the CUSA [68]

	Prevention of cholangitis

Antireflux valve:	*Ando modification*: Better exposition of the biliary remnant, by dividing the ligamentum venosum (Arantius' remnant) [69]
	*Nakajo modification*: Confection of an 2-cm long antireflux valve by invaginating the proximal intestinal portion of the Roux-en-Y limb into its denuded distal portion [75].
Enteric conduit:	*Kasai II-operation*: Creation of a double-Y, where the proximal part of the Roux-en-Y limb is exteriorized through the abdominal wall; the distal segment is end-to-side anastomosed to this proximal segment for continuity of the bile flow [80].
	*Sawaguchi modification*: Complete exteriorization of the entire Roux-en-Y limb as a jejunostomy [81]
	Suruga modification: Double-barrel ostomy of the Roux-en-Y limb [84]
	*Endo modification*: Anastomosis of an ileocolic conduit to the porta hepatis, and exteriorization of the ascending colon as a colostomy including the ileocecal valve [64]
